# Intratumoral Heterogeneity and Longitudinal Changes in Gene Expression Predict Differential Drug Sensitivity in Newly Diagnosed and Recurrent Glioblastoma

**DOI:** 10.3390/cancers12020520

**Published:** 2020-02-24

**Authors:** Ella L. Kim, Maxim Sorokin, Sven Rainer Kantelhardt, Darius Kalasauskas, Bettina Sprang, Julian Fauss, Florian Ringel, Andrew Garazha, Eugene Albert, Nurshat Gaifullin, Christian Hartmann, Nicole Naumann, Sven-Ernö Bikar, Alf Giese, Anton Buzdin

**Affiliations:** 1 Clinic for Neurosurgery, Laboratory of Experimental Neurooncology, Johannes Gutenberg University Medical Centre, Langenbeckstrasse 1, 55124 Mainz, Germany; bettina.sprang@unimedizin-mainz.de (B.S.); jfauss@students.uni-mainz.de (J.F.); 2 Institute of Personalized Medicine, I.M. Sechenov First Moscow State Medical University, 119991 Moscow, Russia; sorokin@oncobox.com (M.S.); mormegill@mail.ru (E.A.); 3 Shemyakin-Ovchinnikov Institute of Bioorganic Chemistry, 117997 Moscow, Russia; 4 Omicsway Corp., 340 S Lemon Ave, 6040, Walnut, CA 91789, USA; garazha@oncobox.com; 5 Clinic for Neurosurgery, Johannes Gutenberg University Medical Centre, Langenbeckstrasse 1, 55124 Mainz, Germany; sven.kantelhardt@unimedizin-mainz.de (S.R.K.); Darius.Kalasauskas@unimedizin-mainz.de (D.K.); Florian.Ringel@unimedizin-mainz.de (F.R.); 6 Department of Pathology, Faculty of Medicine, Lomonosov Moscow State University, 119991 Moscow, Russia; gaifulin@rambler.ru; 7 Department of Neuropathology, Institute of Pathology, Hannover Medical School, 30625 Hannover, Germany; hartmann.christian@mh-hannover.de; 8 StarSEQ GmbH, Joh.-Joachim-Becher-Weg 30a, 55128 Mainz, Germany; naumann@starseq.com (N.N.); Bikar@starseq.com (S.-E.B.); 9 OrthoCentrum Hamburg, Hansastrasse 1, 20149 Hamburg, Germany; alf.giese1@gmail.com; 10 Oncobox Ltd., 121205 Moscow, Russia; 11 Moscow Institute of Physics and Technology, 141701 Moscow Region, Russia

**Keywords:** glioblastoma, glioblastoma stem cells, transcriptomics, recurrent glioblastoma, gene expression, target anti-cancer therapy, molecular pathways

## Abstract

**Background:** Inevitable recurrence after radiochemotherapy is the major problem in the treatment of glioblastoma, the most prevalent type of adult brain malignancy. Glioblastomas are notorious for a high degree of intratumor heterogeneity manifest through a diversity of cell types and molecular patterns. The current paradigm of understanding glioblastoma recurrence is that cytotoxic therapy fails to target effectively glioma stem cells. Recent advances indicate that therapy-driven molecular evolution is a fundamental trait associated with glioblastoma recurrence. There is a growing body of evidence indicating that intratumor heterogeneity, longitudinal changes in molecular biomarkers and specific impacts of glioma stem cells need to be taken into consideration in order to increase the accuracy of molecular diagnostics still relying on readouts obtained from a single tumor specimen. **Methods**: This study integrates a multisampling strategy, longitudinal approach and complementary transcriptomic investigations in order to identify transcriptomic traits of recurrent glioblastoma in whole-tissue specimens of glioblastoma or glioblastoma stem cells. In this study, 128 tissue samples of 44 tumors including 23 first diagnosed, 19 recurrent and 2 secondary recurrent glioblastomas were analyzed along with 27 primary cultures of glioblastoma stem cells by RNA sequencing. A novel algorithm was used to quantify longitudinal changes in pathway activities and model efficacy of anti-cancer drugs based on gene expression data. **Results**: Our study reveals that intratumor heterogeneity of gene expression patterns is a fundamental characteristic of not only newly diagnosed but also recurrent glioblastomas. Evidence is provided that glioblastoma stem cells recapitulate intratumor heterogeneity, longitudinal transcriptomic changes and drug sensitivity patterns associated with the state of recurrence. **Conclusions**: Our results provide a transcriptional rationale for the lack of significant therapeutic benefit from temozolomide in patients with recurrent glioblastoma. Our findings imply that the spectrum of potentially effective drugs is likely to differ between newly diagnosed and recurrent glioblastomas and underscore the merits of glioblastoma stem cells as prognostic models for identifying alternative drugs and predicting drug response in recurrent glioblastoma. With the majority of recurrent glioblastomas being inoperable, glioblastoma stem cell models provide the means of compensating for the limited availability of recurrent glioblastoma specimens.

## 1. Introduction

Glioblastoma (GB), with a median survival rate of around 15 months, ranks amongst the most aggressive of human cancers [1]. The current standard of care for GB consists of de-bulking surgery followed by combined treatments with fractionated ionizing radiation (IR) and the DNA alkylating agent temozolomide (TMZ) [2]. Unfortunately, the overall effectiveness of standard therapy is limited because of the high degree of intrinsic radioresistance and restricted effectiveness of TMZ working in only about a half of GBs [3]. Tumor recurrence after initial treatment is inevitable and poses a major challenge in clinical management of GB. At the stage of recurrence, no effective therapeutic options are currently available, rendering recurrent GB (recGB) a lethal condition. Despite intensive efforts to optimize dosage/combination regimens of cytotoxic monotherapies for recGB, no level one evidence has been achieved so far [4,5,6]. Molecular diagnostic criteria specific for recGB do not exist either. Clinical recommendations for recGB are based on the status of the very same predictive markers as those assessed in the precursor tumor at the time of initial diagnosis. Recent advances indicate that such an assumption may be misleading because it does not take into consideration the longitudinal changes in molecular patterns during GB growth after (or under) cytotoxic treatments. Although the mechanisms of molecular diversification during GB recurrence are still incompletely understood, the existing evidence suggests that alkylating treatments including TMZ may promote proactively the emergence of a hypermutator phenotype [7,8]. Recent advances indicate that so-called therapy-driven molecular evolution is a fundamental trait associated with GB recurrence. Furthermore, there is an emerging consensus that traditional diagnostic approaches based on analyses of a single tumor biopsy may be insufficient for implementing molecular diagnostics of GBs that are notorious for the high degree of intratumoral spatial heterogeneity [9,10,11,12,13]. Reflecting the importance of sampling strategy and treatment-driven molecular evolution in (re)shaping molecular landscapes during GB recurrence, a so-called GLASS (The Glioma Longitudinal AnalySiS, https://www.glass-consortium.org/) consortium has recently been formed to address these issues in the frame of a multicenter investigation [14].

The biological paradigm of GB’s therapeutic resistance is that traditional radiochemotherapy fails to target effectively a specialized fraction of glioblastoma cells, collectively called glioma stem cells (GSCs). According to the hierarchical paradigm of cancer, GSCs constitute the most clinically relevant compartment capable of maintaining tumor growth under cytotoxic treatments [15,16,17,18]. Owing to their inherent cellular plasticity, GSCs may contribute to intratumoral molecular heterogeneity via a non-mutational mechanism based on dynamic interconversions between distinct cellular states as indicated by single-cell transcriptomic analyses [19,20]. While there is a growing sense of importance for considering the impacts of intratumor heterogeneity, longitudinal changes and GSCs in GB progression, there is a paucity of studies addressing these diverse impacts within the same investigation using patient-matched tumor tissues and GSCs.

Furthermore, the interpretation of high throughput omics data is limited by the capacity of computational approaches used for identifying predictive biomarkers of therapeutic response [21,22,23]. RNA sequencing (RNA-seq) has become a gold standard in the field of gene expression studies [24,25]. Both individual gene expression and molecular pathway activation levels can be used for tumor diagnosis and prognosis of treatment outcomes [26,27,28,29]. However, the activation level of molecular pathways provides more robust biomarker signatures compared to individual genes [30,31]. Based on pathway activation analysis and expressions of drug target genes, an approach was proposed that makes it possible to personalize the ranking of anticancer target drugs (ATDs) for an individual tumor [27,32,33]. This approach was recently successfully applied for several cases of off-label prescriptions of ATDs to patients with advanced or metastatic solid tumors [27,34,35,36].

In this study, a combinatorial approach integrating multisampling strategy and longitudinal and complementary transcriptomic investigations was undertaken to identify transcriptomic traits associated with recGB in whole-tissue specimens of GB and tumor-matched GSCs.

## 2. Results

### 2.1. Experimental Samples and Study Design

**Figure 1:**
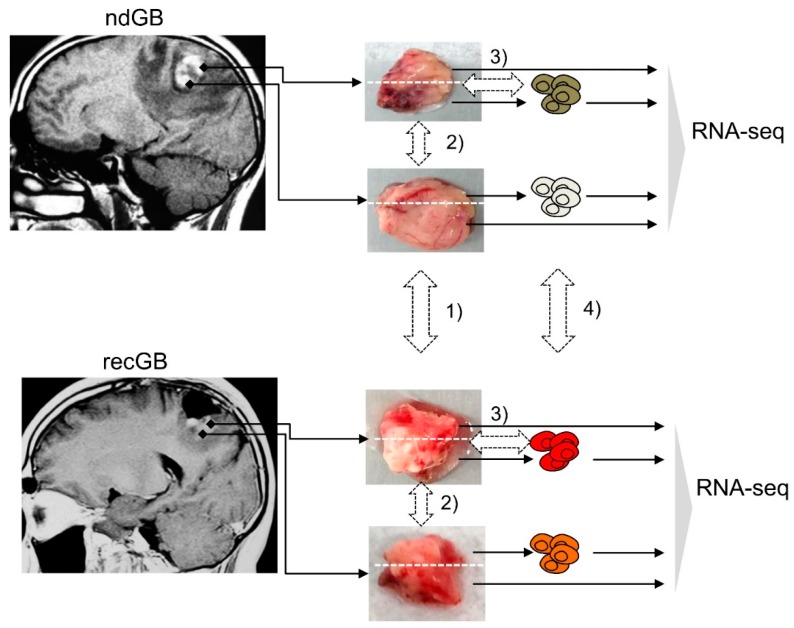
Study design. Tissue blocks were resected from newly diagnosed glioblastomas (ndGBs) or recurrent GBs (recGBs) using a multisampling approach. Each block was portioned and processed either for a direct sequencing of tissue RNA or glioma stem cell (GSC) isolation. RNA-seq of tissue samples or primary cultures of GSCs was followed by bioinformatic analysis to compare gene expression along the following lines: (1) ndGB vs. recGB tissues; (2) between different regions from the same tumor; (3) tissue biopsies vs. GSCs; (4) GSCs from ndGBs vs. GSCs from recGBs.

For RNA-seq, tumor specimens were collected from 28 patients with GB (14 males, 14 females, mean age 55.6 and 59.6 years old, respectively), listed in Supplementary Table S1. From a total of 44 tissue specimens analyzed in this study, 23 were newly diagnosed GBs (ndGBs), 19 were recurrent GBs (recGBs) and two were secondary recurrent GBs. For the 19 ndGBs and 11 recGBs, multiple samples of the same tumor were analyzed by RNA-seq. In 14 cases, matched tumor samples could be obtained from the same patient at ndGB and recGB stages (Supplementary Table S1). From the 14 patient-matched tumors, 12 ndGBs and eight recGBs were represented by more than one sample (Supplementary Table S1). From the nine ndGBs and four recGBs, short-term cultures of glioma stem-like cells (GSCs) could be established and analyzed by RNA-seq. Computational analysis of RNA-seq data encompassed differential expression analysis at gene- and pathway-levels along the following lines: (1) ndGB vs. recGB tissues; (2) between different regions from the same tumor; (3) tissue biopsies vs. GSCs; (4) GSCs from ndGBs vs. GSCs from recGBs (Figure 1).

### 2.2. Quality Assessments of RNA-seq Data

Technical quality of primary RNA-seq data was checked using Illumina SAV software. The FASTQ files were then aligned using the STAR aligner. Expression levels were determined for 26,228 human genes with the unique Human Genome Organization (HUGO) Gene Nomenclature Committee HGNC identifiers. The sequencing profiles obtained were deposited in the Gene Expression Omnibus (GEO) repository under accession number GSE139533. The detailed quality analysis for all experimental transcriptome profiles is shown in Supplementary Table S2. The data obtained were fully methodologically compatible with our previously published ANTE (Atlas of Normal Tissue Expression) atlas of normal brain human tissue RNA-seq profiles [37].

### 2.3. Multisampling Approach Reveals a High Degree of Intratumoral Diversity of Transcriptomic Patterns in ndGBs and recGBs

**Figure 2:**
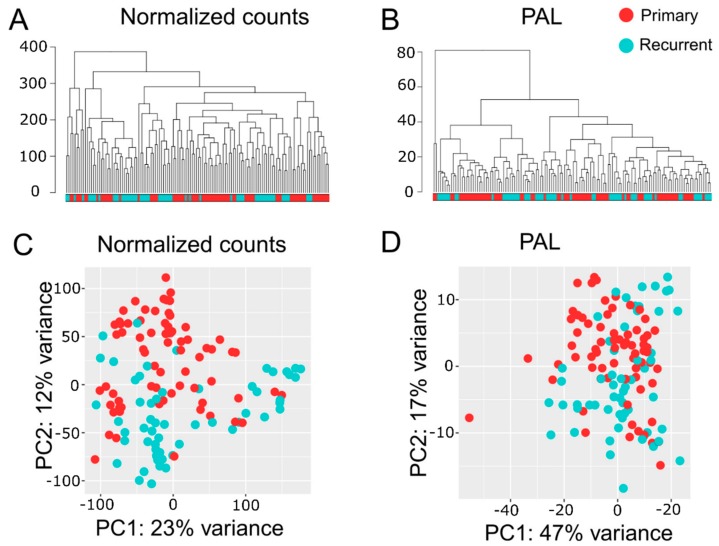
Hierarchical clustering dendrogram of RNA-seq profiles for GB tissue samples. Normalized gene expression (**A**) and pathway activation level (PAL, **B**) data were used to calculate Euclidian distance between the samples. Principal component (PC) analysis of RNA-seq profiles for GB tissue samples using normalized gene expression (**C**) or PAL (**D**) data. Color marker in PCA plots indicates tissue type. Values aligned with axis show proportion of variance in percent for principal components 1 and 2, respectively. Color marker indicates tissue type (ndGB or recGB).

**Figure 3:**
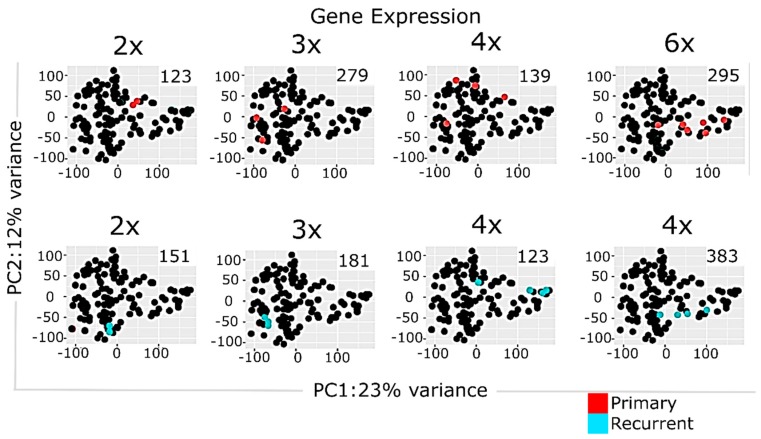
Principal component analysis of multisampled GB tissues using normalized gene expression data. Panels represent, respectively, colored data for individual tumors. Number of profiled samples is indicated. Color marker indicates tissue type (ndGB or recGB). Values aligned with axis show proportion of variance in percent for principal components 1 and 2, respectively.

Transcriptome analyses encompassed two levels, namely differential gene expression and quantification of molecular pathway activities using a recently developed bioinformatic method Oncobox [36,38]. In total, we identified 1903 differentially expressed genes for ndGB and recGB tissues (Supplementary Table S3). Hierarchical clustering revealed mixed patterns in ndGBs and recGBs at the level of either individual transcripts or pathways activities (Figure 2A,B). A principal component analysis (PCA) also revealed a considerable overlap between ndGB and recGB transcriptomes (Figure 2C,D). To exclude the impacts of inter-patient variations, we narrowed our analyses to a subset of matched samples of tumors resected at ndGB and recGB stages from the same individual. PCA analyses revealed varying degrees of co-clustering between ndGB and recGB samples at gene and pathway levels (Supplementary Figure S1). In most cases, ndGB and recGB profiles were broadly dispersed, indicating that the spatial transcriptomic divergence is a common feature in ndGBs and recGBs (Supplementary Figure S1). There was a clear dependency between the number of profiles per tumor and discernibility of intratumoral transcriptomic heterogeneity in ndGBs and recGBs (Figure 3). This suggests that sampling bias may significantly confound interpretations of profiling data when obtained from a single specimen per tumor.

### 2.4. Multi-Sampled Approach Does Not Reveal the Prevalence of Mesenchymal Signature in recGBs

Previous analyses of transcriptomic alterations associated with recGB suggested a shift from *Proneural* to *Mesenchymal* signature detected in a subset of recGBs [39]. To test this conclusion based on analyses of single tumor specimens [39] we aligned gene expression profiles generated from multi-sampled specimens in this study against the major signatures associated with clinically distinct molecular subtypes of GB [39]. The results showed that both the *Proneural* and *Mesenchymal* subtypes were represented abundantly also in our sample collection (Supplementary Figure S2). However, we found no correlation between a particular subtype (*Proneural* or *Mesenchymal*) and tumor stage (ndGB or recGB). Furthermore, in 18% of the cases different samples derived from different regions of the same tumor could be assigned to different (*Proneural* and *Mesenchymal)* subtypes (Supplementary Figure S2).

In order to test whether the observed transcriptomic heterogeneity is connected with tumor size we calculated tumor size as a sum of two tumor dimensions, as shown in Supplementary Table S1. We compared individual tumors size with the median size of all tumors analyzed and applied chi-square goodness of fit test to determine whether the proportions of tumors either bigger or smaller than the median size (Supplementary Figure S2A, blue and yellow bars), differed between *Proneural* and *Mesenchymal* subgroups. The results showed that the difference was not significant (*p*-value 0.72) and that tumors belonging to either *Proneural* and *Mesenchymal* subtypes were not associated with differences in tumors sizes.

### 2.5. recGB-derived GSCs Retain Transcriptomic Patterns Associated with GB Recurrence

**Figure 4:**
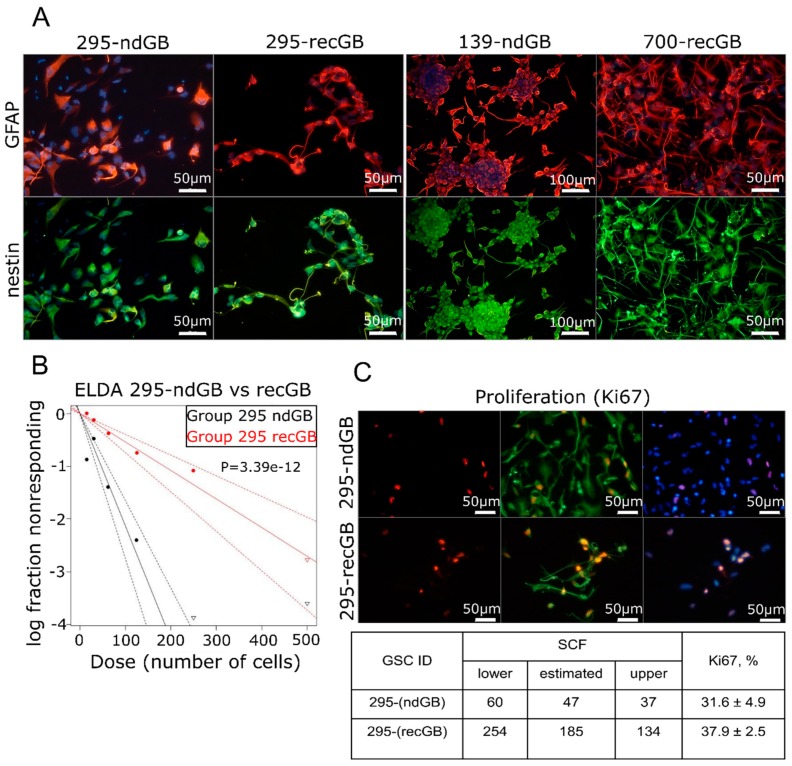
Assessments of stemness attributes and proliferation in GSCs. (**A**) Expression of the neural stem cell/neural progenitor marker nestin (green) and astrocytic marker GFAP (red) in GSCs isolated from ndGBs (samples 295-ndGB and 139-ndGB) or recGB (samples 295-recGB and 700-recGB). Samples 295-ndGB and 295-recGB represent isogenic GSCs derived from the same patient at the time of initial diagnosis or at recurrence, respectively. (**B**,**C**) Quantitative analyses of the self-renewal activity by ELDA (B, representative image) and proliferation rate by assessing Ki67 expressing cells using immunofluorescence staining (C, representative image). The table summarizes quantification results of ELDA and Ki67. “SCF”, stem cell frequency; “lower” and “upper”, confidence intervals for 1/(stem cell frequency). 295-ndGB and 295-recGB correspond to isogenic GSCs derived from the same patient at the time of initial diagnosis or at recurrence, respectively.

**Figure 5:**
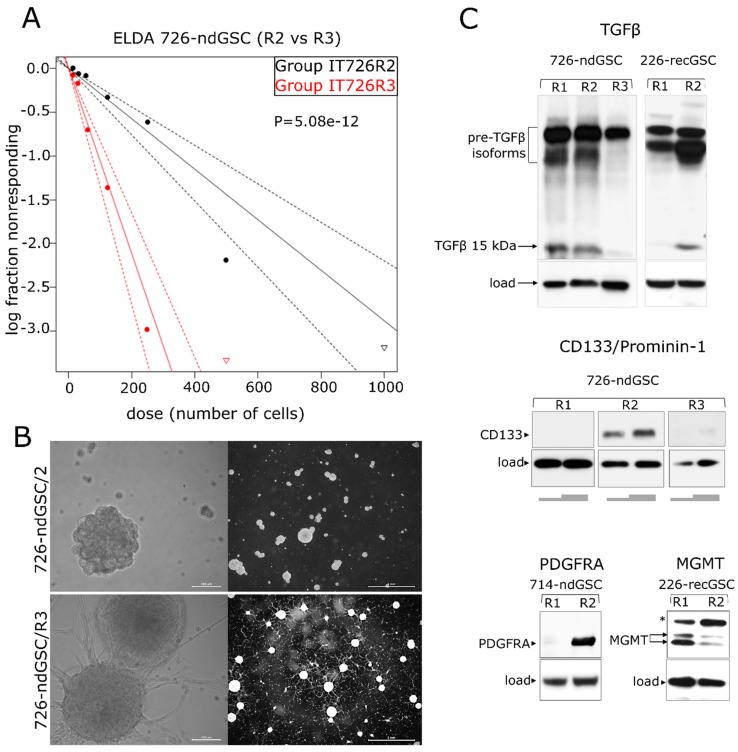
Varying degrees of stemness and diversity of gene expression patterns among isogenic GSCs. (**A**,**B**) GSCs isolated from different regions (designated as R2 or R3) of the same tumor (ndGB #726) differ significantly (*p* = 5.08 × 10^−12^) in the degree of self-renewal (A, graphical presentation of ELDA results) and cellular morphology (B, phase contrast microscopy using objective lens magnifications 20× and 1.6×, scale bars indicated); “log fraction nonresponding” indicates frequency of cells uncapable of forming clonal spheres, “dose (number of cells)” indicates number of cells per mL. The data values with zero negative responses are represented by down-pointing triangles. The dotted lines give 95% confidence interval. (**C**) Variable expression of TGFβ (top), CD133 (middle), PDGFRA and MGMT (bottom) in GSCs derived from different regions of the same tumor (designated as R1, R2 or R3). Grey bars beneath the “CD133” panel correspond to the varying amounts of cell lysate loaded per well. Arrows indicate major isoforms of the proteins analyzed. “*” indicates an abnormally migrating MGMT isoform expressed in GSCs. 726-ndGSC and 226-recGSC correspond to isogenic GSCs obtained from ndGB or recGB, respectively.

Intratumoral diversity of gene expression patterns poses a challenge to concluding about the association between transcriptomic patterns and GB recurrence after initial therapy. Keeping in mind that GSCs have been implicated as the most clinically relevant cellular target responsible for the therapeutic resistance and recurrence in GB [40], we hypothesized that GSCs may recapitulate transcriptomic patterns associated with recGB. To test this hypothesis, we conducted parallel investigations using GSCs isolated from nine ndGBs and four recGBs, by RNA-seq (Supplementary Table S1). In six cases of ndGBs and three recGB cases, GSC cultures could also be established from different regions of the same tumor. In one case, matched GSCs could be isolated from the same patient at ndGB and recGB stages. GSCs were evaluated for stemness attributes and expression of selected GB-associated factors, as exemplified in Figure 4 and Supplementary Figure S4. Notably, our investigations revealed that in some cases, GSCs originating from different regions of the same tumor differed markedly in the morphology of the cells and self-renewal capacity (Figure 5). For example, isogenic GSCs “IT726R2” and “IT726R3”originating from different regions of ndGB #726 showed significant differences (*p* = 5.08 × 10^−12^) in the number of cells required for generating clonal self-renewing spheres (Figure 5A). Furthermore, there were also profound differences in the steady-state levels of several proteins implicated as therapeutic targets in GB such as glioma promoting factor TGFβ [41,42], TMZ-resistance factor MGMT [43], stemness marker CD133/Prominin-1 [44] or the marker of *Proneural* subtype PDFGRα [45] (Figure 5). These results provide evidence that GSCs recapitulate interregional divergence at the cellular and molecular level.

**Figure 6:**
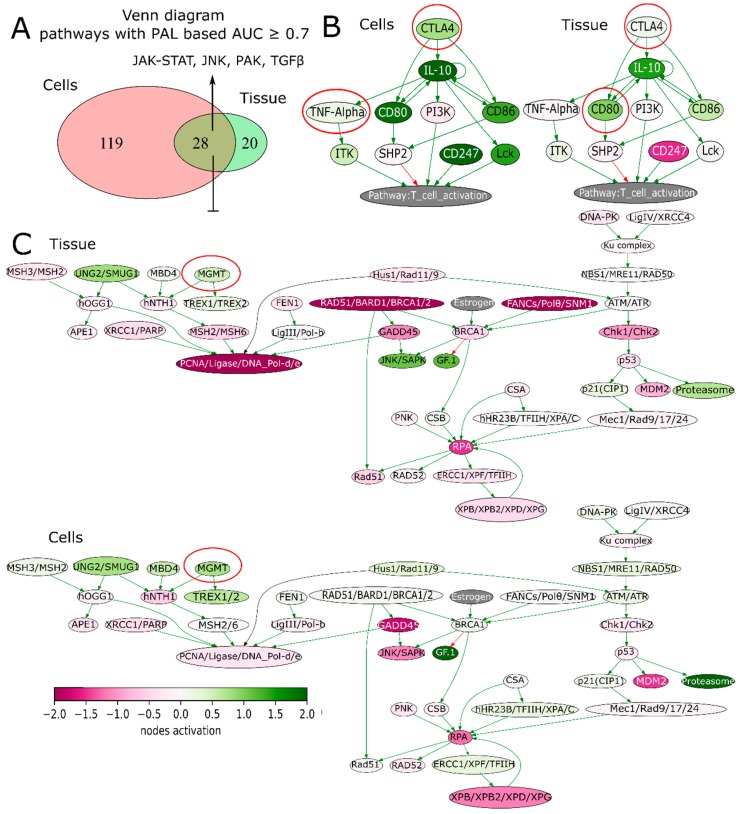
(**A**) Venn diagram illustrating differential molecular pathways in recGB vs. ndGB stages for GSCs and GB tissues. (**B**) Pathway activation profile of the co-stimulatory T-cell signaling pathway in recGB vs. ndGB stages for tissue samples and GSCs. Genes encoding molecular targets for immunotherapeutics (Ipilimumab, Durvalumab) are circled. (**C**) Pathway activation profile of the DNA damage response pathway in recGB vs. ndGB stages for tissue samples and GSCs. Gene *MGMT* is circled.

**Figure 7:**
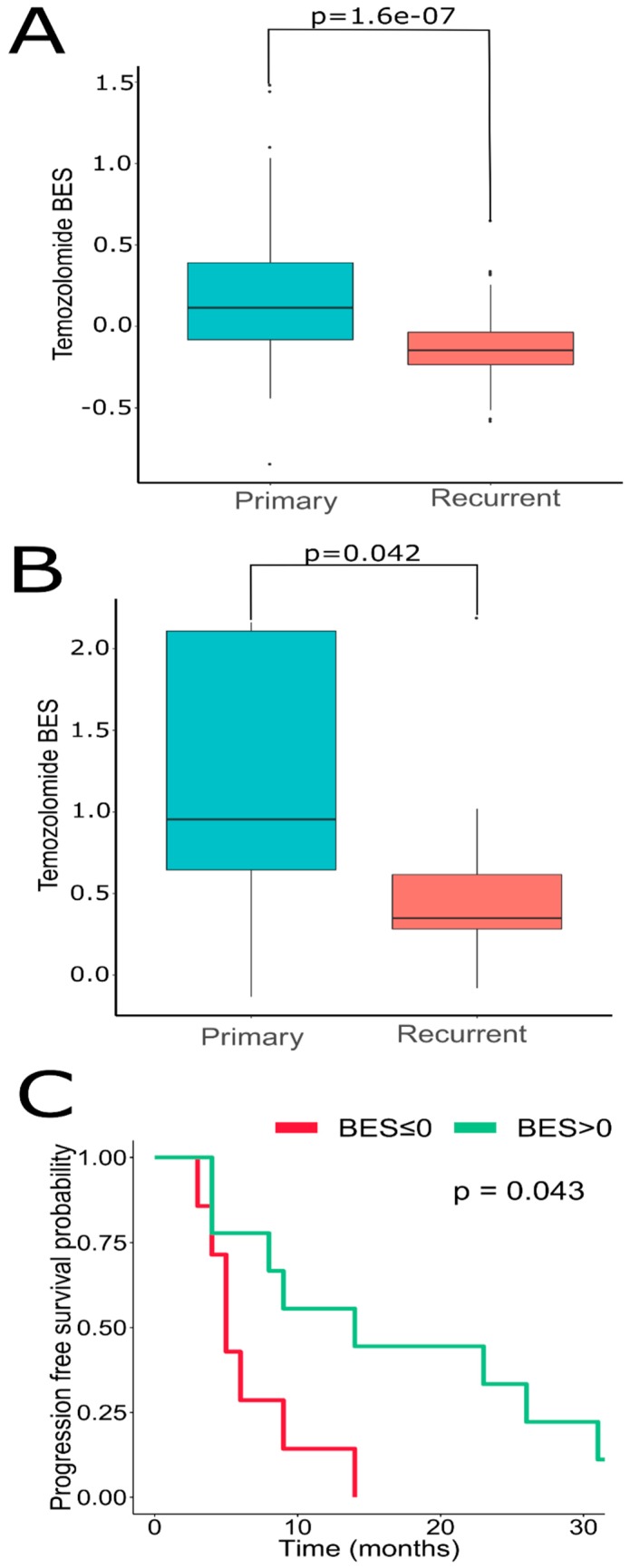
Oncobox BES for TMZ is higher in ndGB vs. recGB sets for both (**A**) GB tissues, Wilcoxon test *p*-value = 1.06 × 10^−7^; and (**B**) GSCs, Wilcoxon *p* = 0.042. (**C**) Dependence of GB patient’s progression-free survival on Oncobox BES for Temozolomide, *n* = 16 patients with ndGB. Hazard ratio = 0.29 (95% CI, 0.088–0.96, *p* = 0.043) for patient level analysis. Hazard ratio = 0.18 (95% CI, 0.078–0.4, *p* < 0.0001) for individual sample level analysis. The analysis was performed using R ggsurvplot package.

We next sought to address if GSCs may capture transcriptomic traits associated with recGBs. To that end, gene expression profiles were determined in GSCs isolated from either ndGBs or recGBs (designated as “ndGSCs” or “recGSCs”, respectively). Similarly to GB tissues (Figure 2), ndGSCs and recGSCs showed no distinct clustering but mixed patterns with ndGSCs and recGSCs grouping together (Supplementary Figure S3A). We asked if some of the differentially expressed genes (DEGs) identified through comparison of ndGB and recGB tissues (Figure 1, comparison line 1) may also be expressed differentially between ndGSCs and recGSCs (Figure 1, comparison line 4). Cross-comparison of both DEG sets identified 370 common genes (Supplementary Figure S3B), supporting the hypothesis that GSCs may recapitulate some of the transcriptomic differences between ndGBs and recGBs. We next compared transcriptomic patterns determined in GB tissues or GSCs at the level of pathways. To identify pathways, differentially active in ndGB and recGB stages transcriptomic data were used to calculate pathway activation levels (PALs) and determine the area under the ROC curve (AUC). By using this approach, 48 pathways from the tissue set and 147 pathways from the GSC set were found to differ significantly in their activities (AUC ≥ 0.7) between ndGB and recGB stages (Supplementary Table S4). Cross-comparison of differentially active pathways revealed 28 common pathways showing the same pattern of change (upregulation or downregulation) in either recGB tissues or recGSCs (Figure 6A and Supplementary Table S4), indicating a high degree of concordance between the results of pathway analyses using GB tissues or GSCs. Notably, several prominent glioma-promoting pathways including TGFβ, PAK, JNK and JAK-STAT [46] and, interestingly, “the co-stimulatory signal during T-cell activation” pathway, were identified among upregulated pathways in both recGB tissues and recGSCs. In contrast, FOXM1 and glucocorticoid receptor (GR) pathways were downregulated in both recGB tissues and recGSCs (Figure 6A,B). Interestingly, both tissue and GSCs analyses revealed upregulation of the MGMT pathway in the state of recurrence (Figure 6C). Considering that MGMT is the major molecular determinant of chemoresistance to TMZ in GB [3,43], the latter finding suggests that recGBs may be less responsive to TMZ than ndGBs. To validate this assumption, we modelled the responsiveness to TMZ by calculating a metric termed the balanced efficiency score (BES), which is a numeric measure of drug response that can be predicted using expression levels of genes, the function of which is essential in determining tumor sensitivity to a given drug [47]. Demonstrating the adequacy of the BES ranking approach, a subset of ndGBs for which the information on progression free survival (PFS) was available (*n* = 16) showed a statistically significantly association between BES–TMZ > 0 and increased PFS (HR = 0.29, 95% CI: 0.088–0.96, *p* = 0.043) (Figure 7C). To compare BES–TMZ between ndGBs and recGBs, BES–TMZ scores were calculated from gene expression data obtained with GB tissues or GSCs. In both tissues (Figure 7A) and GSC sets (Figure 7B), the median BES–TMZ value was found to be significantly (*p* < 0.05) lower in the stage of recurrence than in the ndGB stage. These results suggests that effectiveness of TMZ in recGBs might be lower than in ndGBs, a prediction agreeing with the results of clinical studies showing lack of significant benefit from TMZ in patients with recGB [5]. We next applied the BES ranking to model the efficacy of other anticancer target drugs (ATDs) with known primary molecular targets. In both GB tissues and GSC sample sets, several ATDs were predicted to have differential efficacies at recGB and ndGB stages. Notably, changes in BES values suggest that ndGBs and recGBs may differ in their sensitivity to the same drugs. For example, immunotherapeutic drugs Durvalumab and Ipilimumab currently tested in several clinical trials on recGB (NCT02794883, NCT03707457, NCT02017717) were predicted to be more effective against recGBs than ndGBs in our analyses (Table 1). The simulated efficacy of immunotherapeutic ATDs is also congruent with the observed pattern of activation of “the co-stimulatory signal during T-cell activation” molecular pathway (Figure 6B). In contrast, BES scores for Lomustine and TMZ, which are first-line chemotherapy for ndGB, were lower in recGBs than ndGBs (Table 1), in line with the lack of significant therapeutic effect of alkylating treatments in patients with recGB [5].

## 3. Discussion

In this study, a multilevel approach was implemented to gain insights into the transcriptomic patterns associated with GB recurrence. The major novelty of our study is that it integrates a multisampling approach, longitudinal transcriptomic profiling and complementary investigations using GB tissues and GSCs in the frame of the same investigation. The approach undertaken in our research meets state-of-the-art requirements outlined in the conceptual paper by the international consortium GLASS, which has emphasized the importance of considering the impact of intratumoral spatial diversity and longitudinal changes in molecular diagnostics for gliomas [14].

We provide evidence that intratumor transcriptomic heterogeneity is an intrinsic characteristic conserved in ndGBs and recGBs. Our results confirm and extend the conclusion reached from previous studies in ndGBs [9,11,48,49] that gene expression signatures associated with clinically distinct molecular subtypes of GBs may co-exist within the same tumor at both ndGB and recGB stages of the disease. Interestingly, although the *Proneural* and *Mesenchymal* signatures were identified also in our collection of ndGBs and recGBs, our data do not support the previously proposed hypothesis that a shift from the *Proneural* toward the *Mesenchymal* signature is a transcriptomic trait associated with GB recurrence [39,45]. One possible explanation for this discrepancy is the difference in sampling strategies. In previous studies, transcriptome analyses and comparison between ndGBs and recGBs were conducted using single samples of patient-matched tumors, whereas in our study, all but one patient-matched tumors were represented by more than one tissue sample. Profiling of multiple samples in our study has enabled to reveal that assignment of a particular tumor to a particular molecular subclass can be influenced by the number of profiled samples (Figure 3 and Figure 5). Our data suggest that intratumor heterogeneity of transcriptomic patterns may be contributed by GSCs in ndGBs and recGBs. We provide the first direct evidence that GSCs derived from different regions of the same tumor may differ markedly in the expression levels of pleiotropic factors playing key roles in defining the transcriptional and cellular landscapes in GBs (TGFβ, PDGFRA, CD133, Figure 5). Collectively, our data lead us to conclude that spatial variations in gene expression may be a confounding variable in transcriptome-association studies, especially for the longitudinal tracking of transcriptomic changes during GB recurrence.

An important question following from this conclusion is, which sampling approach might be most adequate or feasible to provide sufficient accuracy in predicting treatment responses in GBs? Based on the results of genome-wide mutational analyses, it was calculated that on average as many as four biopsies would be required to provide a 50% chance of identifying at least 80% of mutational alterations in GBs [9]. Recent studies indicate that increasing the number of samples per se may not be the solution because the distribution of distinct transcriptional patterns within the tumor is not random but determined by the intratumoral architecture of GBs [49]. Furthermore, implementing multi-sampling as a standard approach to transcriptome diagnostics of GBs is challenged by practical and conceptual issues. On the practical side, there is an issue of feasibility of practical implementation of multi-sampling in clinical practice. Especially for recGBs, most of which (~70%) are inoperable [5] and therefore inaccessible for the systematic profiling of tumor tissues. Apart from practical issues, there is also a conceptual hurdle related to the lack of certainty about the biological and clinical meaningfulness of different transcriptomic patterns co-existing within the same tumor. In this regard, our finding that recGSCs capture multiple transcriptomic traits associated with recGBs suggests a potential solution to existing impediments. Our results indicate that comparative profiling of ndGSCs vs. recGSCs represents an adequate approach to define the longitudinal transcriptome profile of recGBs.

Of special interest is our finding that recGB tissues show transcriptome changes that are consistent with the augmentation of the MGMT pathway, the major biomarker of chemoresistance to TMZ in GB [3,43]. Our findings are concordant with predictions drawn from comparative genomic profiling that sensitivity to TMZ in recGBs might be even lower than in ndGBs [8,50]. Our study provides novel transcriptomic evidence indicating that loss of sensitivity to TMZ may be a universal trait associated with GB recurrence. However, this hypothesis based on biomathematical analyses still needs to be proven experimentally. The most straightforward approach to address this hypothesis would be direct testing of the therapeutic response to TMZ in tumors driven by recGB-derived GSCs. This information would be essential for interpreting the data on GSC sensitivity to TMZ in vitro, especially considering that GSC responses in vitro may not necessarily reflect their responses in the tumor context. For example, assessments of self-renewal capacity used to evaluate GSC proliferation/vitality in vitro may not be an adequate surrogate measure for evaluating the therapeutic response and tumor-propagating capacity [51]. While providing a transcriptional rationale for empirical clinical evidence for the lack of significant therapeutic benefit from TMZ in patients with recGB [5], our results predict that Durvalumab and Ipilimumab may be more effective than alkylating agents in the context of recGBs. Such prediction is concordant with the therapeutic paradigm based upon the concept of immune checkpoint inhibition in recGBs [6].

## 4. Materials and Methods

### 4.1. GB Tissue Samples

One hundred twenty-eight tumor samples were collected from 28 patients with ndGB or recGB operated at the Johannes Gutenberg University Medical Center Mainz (UMM). Written informed consents for using excess tumor tissue for research purposes were obtained from all the patients. Resected excess tumor tissue samples were processed within 40 min after resection and divided into three portions under a sterile hood. One portion was snap-frozen in liquid nitrogen and kept at −80 °C for RNA-seq after evaluation by a neuropathologist. Second portion was used for histopathological assessments. Third portion was used for isolating GSCs. Tumor samples and GSCs were coded and processed for RNA-seq anonymously and in accordance with the approval by the UMM Institutional Review Board and ethics committee approval No. 837.178.17(11012) granted to the UMM Clinic for Neurosurgery by the Rhineland Palatinate Chamber of Physicians (Landesäzrtekammer Rheinland-Pfalz, https://www.laek-rlp.de/ausschuesse-kommissionen/ethikkommission/).

### 4.2. Cell Cultures

GSCs were isolated from freshly resected tumor tissue using a brain tissue dissociation kit (P) (Miltenyi Biotec GmbH, Bergisch Gladbach, Germany). Single cell suspensions were maintained in NeuroBasal medium supplemented with the B27 component (Invitrogen, Baden-Württemberg, Germany), human fibroblast growth factor (FGF, 10 ng/m) and epidermal growth factor (EGF, 20 ng/mL) (Biochrom GmbH, Berlin, Germany). Self-renewal capacity was evaluated by using the extreme limited dilution assay [52]. In brief, after dissociation, vital cells were seeded in 24-well plates at a defined density, typically from 100 to 1000 cells/mL and incubated for 4–6 weeks to allow for clonal spheres formation. Spheres were counted under microscope. Stem cell frequency was calculated using ELDA software [52]. For immunofluorescence staining, 20,000–30,000 cells were seeded on glass coverslips pre-coated with poly-L-ornithine hydrobromide (15 µg/mL, Sigma Aldrich, Munich, Germany) and cultured for indicated time points. Cells were fixed with 4% paraformaldehyde/PBS (Merck KGaA) for 5 min at RT followed by methanol/acetone (50% *v*/*v*) fixation at −20 °C overnight. Cell permeabilization was performed using 0.3% Triton X-100/PBS (Sigma) for 5 min at RT. Primary antibodies used in the study included α-nestin (Abcam ab22035), α-GFAP (Dako Z0334) and secondary antibodies (goat α-mouse Alexa Fluor 488 or goat α-rabbit Alexa Fluor 555; Invitrogen, Baden-Württemberg, Germany).

### 4.3. Preparation of Libraries and RNA Sequencing

Frozen tumor samples were routinely homogenized using the Minilys personal homogenizer and the Precellys Evolution homogenizer (Bertin Instruments, Frankfurt am Main, Germany). Grinding was performed using a combination of 1.4 mm and 2.8 mm ceramic beads and 20 mg of tumor tissue at 6500 rpm for 30 s × 3 and 30 s × 2 pauses (4 °C). RNA extraction was carried out using the Precellys Tissue RNA Kit Safety-Line (Peqlab) following the manufacturer’s protocol. Then, RNA integrity number (RIN) was measured using an Agilent 2100 Bioanalyzer using an Agilent RNA 6000 pico and nano assay. RNA concentration was measured with Qubit 2 and Qubit 4 fluorometers (Invitrogen, Baden-Württemberg, Germany) using the RNA BR and HS assay kits. Samples with RIN values < 7 were excluded from subsequent library preparation. For library preparations, we used the TruSeq^®^ Stranded Total RNA Library Prep (Illumina^®^, Berlin, Germany) and the NEBNext^®^ Ultra II Directional RNA Library Prep Kit (New England BioLabs^®^, Frankfurt am Main, Germany) according to the manufacturer’s recommendations. Different dual index adaptors were used for multiplexing samples in one sequencing run. Library concentrations and quality were measured using a Qubit dsDNA high-sensitivity (HS) kit and QIAxcel capillary electrophoresis system with QIAxcel ScreenGel software (QIAGEN GmbH, Hilden, Germany). RNA sequencing was performed at StarSEQ using Illumina NextSeq 500 equipment for paired-end sequencing, 150 bp read length, for approx. 25–30 million raw reads per sample. A data quality check was done on an Illumina Sequencing Analysis Viewer and FastQC. De-multiplexing was performed with Illumina bcl2fastq2 v 2.17–2.20 software (Illumina^®^, Berlin, German).

### 4.4. Primary Processing of RNA Sequencing Data

RNA-seq FASTQ files were first processed with STAR aligner [53]. In total, expression levels were established for 23,582 annotated genes with corresponding HGNC identifiers. For gene expression clustering analyses, the gene expression data were merged into a single dataset and normalized with DESeq2 [54]. Hierarchical clustering was performed using the R ward.D2 method. The dendrograms were visualized with custom R script. Principal component analysis (PCA) was performed with package made4 in R.

### 4.5. Analysis of Molecular Pathway Activation

Pathway activation level (PAL) values were calculated according to Borisov et al. [38] using the formula

PAL_p_ = ∑_n_ NII_np_ ARR_np_ log CNR_n_/∑_n_ |ARR_n_| (1)

where PAL_p_ is the molecular pathway *p* activation level; CNR_n_ (case-to-normal ratio) is the ratio of the protein-encoding gene *n* product concentrations in the test sample and in the norms (average value in the control group); ln is the natural logarithm; NII_np_ is the index of gene product *n* assignment to molecular pathway *p*, assuming the values equal to one for gene products included in the pathway and equal to zero for gene products not included in the pathway; discrete value *ARR_np_* (activator/repressor role) is calculated algorithmically and deposited into the molecular pathway base [38]. ARR for gene *n* in pathway *p* is determined as follows:

$$ARR_{np}=\left\{\begin{array}{l} \begin{array}{l} -1;\mathrm{protein}\;n\;\mathrm{is}\;a\;\mathrm{signal}\;\mathrm{repressor}\;\mathrm{in}\;a\;\mathrm{pathway}\;p\\ -0,5;\;\mathrm{protein}\;n\;\mathrm{is}\;\mathrm{more}\;\mathrm{likely}\;a\;\mathrm{signal}\;\mathrm{repressor}\;\mathrm{in}\;a\;\mathrm{pathway}\;p \end{array}\\ 0;\mathrm{the}\;\mathrm{role}\;\mathrm{of}\;a\;\mathrm{protein}\;n\;\mathrm{in}\;a\;\mathrm{pathway}\;p\;\mathrm{is}\;\mathrm{either}\;\mathrm{ambivalent}\;\mathrm{or}\;\mathrm{netral}\;\;\\ 0,5;\;\mathrm{protein}\;n\;\mathrm{is}\;\mathrm{more}\;\mathrm{likely}\;a\;\mathrm{signal}\;\mathrm{activator}\;\mathrm{in}\;a\;\mathrm{pathway}\;p\\ 1;\mathrm{protein}\;n\;\mathrm{is}\;a\;\mathrm{signal}\;\mathrm{activator}\;\mathrm{in}\;a\;\mathrm{pathway}\;p \end{array}\right\}.$$

In total, 328 molecular pathways having the highest mutation burden in cancers [55,56] were accumulated in the Oncobox pathways database that covered 5611 human protein-coding genes. For generation of the Oncobox database, open access pathway catalogues were used [57,58,59,60].

### 4.6. In Silico Modeling of Drug Efficiencies

Efficiencies of anticancer target drugs (ATDs) were simulated based on individual gene expression profiles according to Tkachev et al. using as a metric the balanced efficiency score (BES) calculated for every ATD [47].

### 4.7. Data Records

Gene expression profiles were deposited in Gene Expression Omnibus database (GEO) under accession number GSE139533. The data is provided as a matrix of raw counts as produced by STAR aligner. Combined meta-information for GBM gene expression profiles used is given in Supplementary Table S1.

## 5. Conclusions

In conclusion, spatial diversity of gene expression patterns is an intrinsic characteristic of GB conserved in ndGBs and recGBs and recapitulated by GSCs. Delineation of transcriptomic traits associated with recGB requires consideration of the impacts of longitudinal changes and intratumoral transcriptome heterogeneity. Profiling of a single sample is insufficient to provide a sufficient level of predictive accuracy and empower molecular diagnostics of GBs. Biologically and clinically relevant criteria need to be delineated in order to implement multisampling approaches in precision diagnostics.

## Figures and Tables

**Table 1:** Anticancer target drugs (ATDs) showing differential (AUC ≥ 0.7) ranking in ndGB vs. recGB comparisons in both tissue-based and GSC-based RNA sequencing profiles.

ATD	BES ndGBs/recGB	BES ndGB-GSCs/recGB-GSCs
Alitretinoin	−4.157/−1.704	−13.694/−12.229
Durvalumab	0.31/0.59	−0.464/0.007
Ibrutinib	6.924/10.173	−18.909/−16.301
Ipilimumab	0.498/1.069	−0.957/−0.806
Lomustine	0.236/−0.115	1.283/0.553
Pomalidomide	7.418/15.207	−21.075/−15.44
Temozolomide	0.236/−0.115	1.283/0.553
Thalidomide	12.289/28.501	−46.919/−39.564
Venetoclax	−2.225/−0.415	−13.04/−12.056

## Supplementary Materials

The following are available online at https://www.mdpi.com/2072-6694/12/2/520/s1, Figure S1. Principal component analysis of GBM tissue multisampling using normalized gene expression data (left panel) or pathway activation levels (right panel). Panels show colored data for patient-matched tumors at ndGB (red) and recGB (magenta) stages. Values aligned with axis show proportion of variance in percent for principal components 1 and 2, respectively; Figure S2. (A) Hierarchical clustering dendrogram of GB tissue samples. Color marker indicates tissue type (ndGB or recGB, for horizontal codes) or phenotype associated genes (PN—proneural, MES—mesenchymal, PROLIF—proliferative) according to Phillips et al. [39]. (B) Hierarchical clustering dendrogram of GB tissue samples adopted from Phillips et al. [39]; Figure S3. Differential gene expression analysis in GSCs. (A) Hierarchical clustering dendrograms of GSC samples. Left panel, clustering by Euclidian distance for all (26,228) genes. Right panel, clustering by Euclidian distance for 1827 genes expressed differentially in recGSCs and ndGSCs. (B) Venn diagram representing intersection of differential genes in ndGBs vs. recGBs for tissues and GSCs; Figure S4. Experimental platform for GSC characterization in vitro. Western blot analysis of transcription factors p53 and ets-1, key regulators of growth/proliferation/invasion signaling in gliomas and MGMT, the major molecular determinant of TMZ resistance in GSCs isolated from ndGBs or recGBs. Arrows indicate major isoforms of the proteins analyzed. “*” indicates an abnormally migrating MGMT isoform expressed in GSCs. Numeric codes correspond to GSCs obtained in this study. Glioblastoma cell lines U87MG and LN229-MGMT with the known p53, Ets-1 and MGMT status were used as a reference. Table S1. Patient data and biosamples analyzed in this study.

## Abbreviations

TMZ	temozolomide
GB	glioblastoma
recGB	recurrent glioblastoma
ndGB	newly diagnosed glioblastoma
GEO	Gene Expression Omnibus
GSCs	glioma stem cells
ATDs	anticancer target drugs
BES	balanced efficiency score
